# The Relationship between Species Diversity and Genetic Structure in the Rare *Picea chihuahuana* Tree Species Community, Mexico

**DOI:** 10.1371/journal.pone.0111623

**Published:** 2014-11-06

**Authors:** Sergio Leonel Simental-Rodríguez, Carmen Zulema Quiñones-Pérez, Daniel Moya, Enrique Hernández-Tecles, Carlos Antonio López-Sánchez, Christian Wehenkel

**Affiliations:** 1 Forestry and Wood Industry Institute, Universidad Juárez del Estado de Durango, Durango, Mexico; 2 Higher Technical School of Agricultural and Forestry Engineering, Universidad Castilla-La Mancha, Albacete, Spain; University of Innsbruck, Austria

## Abstract

Species diversity and genetic diversity, the most basic elements of biodiversity, have long been treated as separate topics, although populations evolve within a community context. Recent studies on community genetics and ecology have suggested that genetic diversity is not completely independent of species diversity. The Mexican *Picea chihuahuana* Martínez is an endemic species listed as “Endangered” on the Red List. Forty populations of Chihuahua spruce have been identified. This species is often associated with tree species of eight genera in gallery forests. This rare *Picea chihuahuana* tree community covers an area no more than 300 ha and has been subject of several studies involving different topics such as ecology, genetic structure and climate change. The overall aim of these studies was to obtain a dataset for developing management tools to help decision makers implement preservation and conservation strategies. However, this unique forest tree community may also represent an excellent subject for helping us to understand the interplay between ecological and evolutionary processes in determining community structure and dynamics. The AFLP technique and species composition data were used together to test the hypothesis that species diversity is related to the adaptive genetic structure of some dominant tree species (*Picea chihuahuana*, *Pinus strobiformis*, *Pseudotsuga menziesii* and *Populus tremuloides*) of the *Picea chihuahuana* tree community at fourteen locations. The Hill numbers were used as a diversity measure. The results revealed a significant correlation between tree species diversity and genetic structure in *Populus tremuloides*. Because the relationship between the two levels of diversity was found to be positive for the putative adaptive AFLP detected, genetic and species structures of the tree community were possibly simultaneously adapted to a combination of ecological or environmental factors. The present findings indicate that interactions between genetic variants and species diversity may be crucial in shaping tree communities.

## Introduction

Species diversity and genetic diversity, the most basic elements of biodiversity, have long been treated as separate topics, although populations evolve within a community context [1], [2]. Recent studies on community genetics and ecology have suggested that genetic diversity is not completely independent of species diversity [3] [4] [5] and may be correlated in three main ways: a) by a parallel process in which simultaneous responses of both levels of diversity to environmental factors may support a positive relationship [4] [6]; b) species diversity may be causally controlled by genetic diversity within component species [7]; and c) genetic diversity may be causally affected by the diversity and relative abundance of coexisting species if the species diversity of a community influences the selection system [8] with a negative association [3].

Although several diversity indices have been described, very few are regularly applied in ecological studies, e.g. the species richness index [9], the Shannon index [10] and the Simpson index [11]. However, many indices can be transformed into members of a family of explicit diversity measures, also known as Hill numbers or family [12] [13] or Rényi-diversity [14] [15] [16] [17].

*Picea chihuahuana* Martínez (Chihuahua spruce) is an endemic species listed as “Endangered” on the Red List and by the Mexican Official Standard [18], [19]. Forty populations of Chihuahua spruce, including about 43,000 individuals, have been identified in three separate clusters in the Sierra Madre Occidental. The clusters occurred at elevations ranging from 2,100 to 3,000 m a.s.l. and with average temperatures between 9 and 12°C [20]. In the Sierra Madre Occidental, Chihuahua spruce grows in areas with precipitation ranging from 600 mm to 1,300 mm [21]. Chihuahua spruce preferentially inhabits areas of rough terrain located on hillsides and canyons in northwest- to northeast-facing areas with slopes ranging from 35% to 80%, at the margins of streams and rivers [22] [23]. This species is often associated with tree species of the genera *Pinus, Quercus, Abies, Pseudotsuga, Populus, Prunus, Juniperus* and *Cupressus* forming gallery forests [20] [22] [24]. This rare pine-spruce-cedar community (hereafter referred to as the *Picea chihuahuana* tree community) covers an area no more than 300 ha. It has remained in its original condition due to its isolated location at high elevations in very rugged mountains [20] [25].

The *Picea chihuahuana* tree community has been the subject of several studies involving different topics such as ecology [20] [22] [26], genetic structure [25] [27] [28] [29] [30] [31] and climate change [32] [65]. The overall aim of these studies was to obtain a dataset for developing management tools to help decision makers to implement preservation and conservation strategies [33].

However, this unique forest tree community may also represent an excellent model for helping us to understand the interplay between ecological and evolutionary processes in determining community structure and dynamics [33] [34]. The Amplified Fragment Length Polymorphism (AFLP) technique and species composition data were used together to test the hypothesis that species diversity is related to adaptive genetic structure of some dominant tree species (*Picea chihuahuana*, *Pinus strobiformis*, *Pseudotsuga menziesii* and *Populus tremuloides*) of the *Picea chihuahuana* tree community at fourteen locations.

## Material and Methods

We confirm that the field studies provide the specific location of study (*Table 1*). No vertebrate studies were carried out. Field permit was granted by SEMARNAT, Mexico (http://www.semarnat.gob.mx/).

**Table 1:** Information about the 14 locations studied in the *Picea chihuahuana* M. tree community.

Code	Location	Property	Municipality	Latitude	Longitude	Elevation
				N	W	m
TN	La Tinaja	Ejido El Ranchito	Bocoyna	27°57′27″	107°46′13″	2,380
RC	El Ranchito	Ejido El Ranchito	Bocoyna	27°57′20″	107°45′12″	2,414
CV	El cuervo	Ejido El Ranchito	Bocoyna	27°57′01″	107°46′18″	2,500
TY	Talayote	Ejido Los Volcanes	Bocoyna	27°55′03″	107°49′01″	2,355
TR	Las Trojas	Ejido El Ranchito	Bocoyna	27°54′27″	107°45′17″	2,395
VN	El Venado	Ejido San Javier	Bocoyna	27°45′41″	107°41′33″	2,311
LQ	La Quebrada	Ejido El Caldillo y su anexo El Vergel	Balleza	26°28′13″	106°21′51″	2,730
PPR	Paraje Piedra Rayada	Ejido Chiqueros	Guanaceví	26°09′15″	106°24′17″	2,600
QD	Quebrada de los Durán	Ejido Chiqueros	Guanaceví	26°08′48″	106°22′53″	2,570
CB	Cebollitas	Private property	Canelas	25°05′55″	106°26′27″	2,450
SJ	San José de las Causas	Ejido San José de las Causas	San Dimas	24°01′07″	105°47′56″	2,480
SB	Santa Bárbara	Ejido El Brillante	Pueblo Nuevo	23°39′44″	105°26′20″	2,725
ACH	Arroyo del Chino	Santa María Magdalena de Taxicaringa	Mezquital	23°21′05″	104°43′05″	2,600
LP	La Pista	Santa María Magdalena de Taxicaringa	Mezquital	23°19′52″	104°45′00″	2,685

### Study area

**Figure 1:**
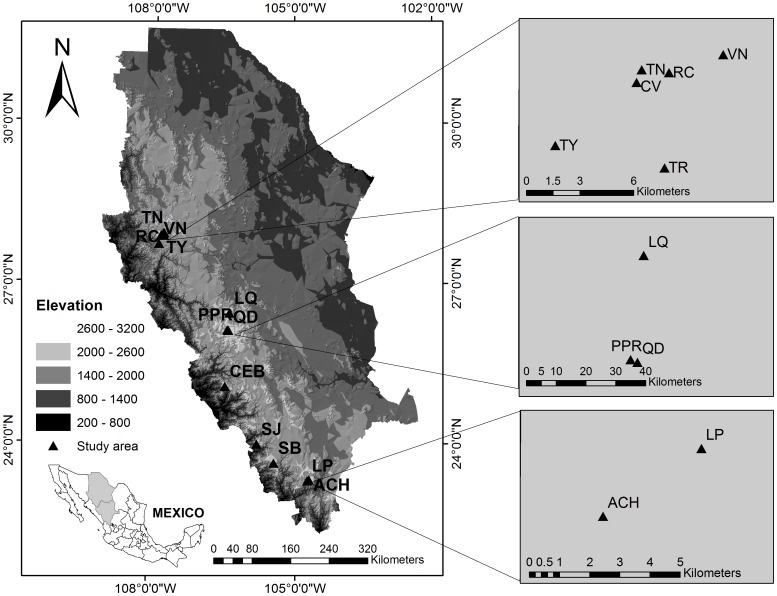
Map of the 14 study locations (black triangles) in the *Picea chihuahuana* tree community, Mexico. 1) La Tinaja (TN), 2) El Ranchito (RC), 3) El Cuervo (CV), 4) Talayote (TY), 5) Las Trojas (TR), 6) El Venado (VN), 7) La Quebrada (LQ), 8) Paraje Piedra Rayada (PPR), 9) Quebrada de los Durán (Arroyo del Indio Ignacio) (QD), 10) Cebollitas (CB), 11) San José de las Causas (SJ), 12) Santa Bárbara (Arroyo del Infierno) (SB), 13) Arroyo del Chino (ACH), and 14) La Pista (Arroyo de La Pista) (LP).

The study area was located in the Sierra Madre Occidental, in the States of Durango and Chihuahua. In order to determine relationship between tree species diversity and genetic structure, fourteen study sites were considered (*Table 1*, *Figure 1*). One plot of 50×50 m (0.25 ha) was established in the center of each site. *Pinus strobiformis*, *Pseudotsuga menziesii* and *Populus tremuloides* were not presented in all plots. For a complete list of the tree species composition and frequencies in the fourteen plots, see Quiñones-Pérez et al. [69].

### Sampling sites

The target tree species *Picea chihuahuana* was fully scored (i.e. all seedlings, saplings and trees were measured). For analysis of the genetic structure, needles were sampled from a total of 194 trees and 475 seedlings and saplings (<7 cm diameter at breast height [*DBH*] from natural regeneration) of *Picea chihuahuana* in the fourteen selected plots (from 17–53 individuals per plot), in 2010–2012. Further investigations were carried out on 44 trees and 85 saplings of *Pinus strobiformis* in twelve plots (from 3–17 individuals per plot), 17 trees and 46 saplings obtained by natural regeneration of *Pseudotsuga menziesii* in six sites (from 10–11 individuals per plot), and 18 trees and 58 saplings from natural regeneration of *Populus tremuloides* in seven sites (from 8–13 individuals per plot) in the *Picea chihuahuana* tree community.

### AFLP analysis

DNA data were obtained by the amplified fragment length polymorphism (AFLP). AFLP fingerprints were established according to the protocol described by Vos et al. [35]. Due to the very large conifer genome, the original protocols were modified for the three conifer species by using a larger number of adaptors (restriction/ligation, 6x) and primers (pre-AFLP and selective AFLP, 6x). The DNA was extracted by use of the QIAGEN DNeasy96 plant kit and digested with the restriction enzymes EcoRI (5′-GACTGCGTACCAATTCNNN-3′) and MseI (5′-GATGAGTCCTGAGTAANNN-3′). PCR amplification was carried out with double-stranded EcoRI and MseI adaptors ligated to the end of the restriction fragments to produce template DNA. In pre-AFLP amplification, the PCR products were treated with the primer combination E01/M03 (EcoRI-A/MseI-G). The reaction was initiated at 72°C for 2 min, followed by 20 cycles each consisting of 94°C for 10 sec, 56°C for 30 sec, and 72°C for 2 min, and a final step at 60°C for 30 min. Selective amplification was carried out with the fluorescent-labeled (FAM) primer pair E35 (EcoRI -ACA) and M70 (MseI-GCT) for *Picea chihuahuana* and the fluorescent-labeled (FAM) primer pair E35 and M63+C (MseI-GAAC) for *Pseudotsuga menziesii*, *Pinus strobiformis*, and *Populus tremuloides*. The fourth selective base was added to reduce the high number of weak signals. The selective PCR cycling started at 94°C for 2 min, followed by 10 cycles, each consisting of 10 sec at 94°C, 30 sec at 65°C and 2 min at 72°C. The 65°C annealing temperature of the first cycle was subsequently reduced by 1°C for the next 10 cycles and continued at 56°C for 30 sec for the remaining 23 cycles, and finished with a final extension step at 60°C for 30 min. All PCR reactions were conducted in a Peltier Thermal Cycler (PTC-200 version 4.0, MJ Research). The amplified restriction products were resolved electrophoretically in a Genetic Analyzer (ABI 3100 16 capillaries), along with the internal size standard GeneScan 500 ROX (fluorescent dye ROX) from Applied Biosystems. The size of the AFLP fragments was determined with the GeneScan 3.7 and Genotyper 3.7 software packages (Applied Biosystems) [70].

Although it is possible to poolplex AFLPs, we have found that the simultaneous analysis of AFLPs sometimes lowers the quality of patterns, leading to problems with scoring. Thus, we used only one primer combination in our first approach with the Mexican tree species under study.

Scoring was fully automated and only strong and high quality fragments were considered. Only fragments above the signal threshold of 50 (minimum peak height) (according to ABI manual) and with a maximum peak width of 1.0, minimum peak size of 75, maximum peak size of 450, tolerance +/− bp of 0.4 and a minimum peak-peak distance of 0.5 were considered.

Quality and reproducibility were checked by reference samples on each plate and independent repetition (replicate PCRs) of at least 16 samples (i.e. minimum 16 individuals per randomly chosen tree species). All replicates showed the same AFLP pattern as in the first analyses, particularly concerning the adaptive loci. Due to slight differences in PCR, the automatic scoring system identified fragments close to the minimum peak height of 50 as different. These fragments were not included in further analyses. To prevent mislabelling similarly sized fragments of different loci as one locus, we checked the adaptive loci manually and found that the size of the fragment varied by less than 0.1.

Finally, four binary AFLP matrices were created from the presence (code 1) or absence (code 0) at potential band positions. Each band detected corresponded to the presence of a dominant genetic variant (plus phenotype) with unknown mode of inheritance of this potential band position (detected fragment length) (named genetic variant ‘1’) [36] [37]. The absence of a band reflected the presence of only recessive genetic (allelic) variants at the given position (locus) (named genetic variant ‘2’). Loci with frequencies of genetic variant <0.05 or>0.95 were excluded from further analyses. Relative frequencies at each AFLP-based locus (*f*) were computed on the basis of the analysis of *Picea chihuahuana, Pinus strobiformis, Pseudotsuga menziesii*, and *Populus tremuloides* specimens from the above-mentioned plots.

### Measuring species diversity and genetic diversity

Considered as a function of *a*, Hill numbers (*ν_a_*) [12], used as diversity measures, describe a variant profile for each frequency distribution. The most illustrative values of the subscript *a* in such a diversity profile are *a* = 0, where the diversity equals the total number of variants, *a* = 2 as the effective number, and *a* = *∞*, where only the most frequent variant determines the diversity (amount of prevalent variant) [13]. *p* is the relative frequency of a variant *i*. Hill numbers can be used as explicit diversity measures at both species and genetic levels of diversity [2] [4]. Formally,
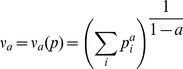
(1)


*p* is the relative frequency of a variant *i*.

To represent the tree species diversity profile (*v_sp,a_*), we selected the described diversities applied to each location. Thus, each location of the *P. chihuahuana* tree community was characterized by the total number of tree species (species richness, (*v_sp,0_*)), effective number of tree species (Simpson index, (*v_sp,2_*)) and the number of prevalent tree species (*v_sp,∞_*) in the sampling plots.

*ν_a_* was also used to calculate the diversity of genetic variants considering only the “effective number” of genetic variants ‘1’ and ‘2’ (*v_g,2_*) at each AFLP locus. A bias correction was carried out for *v_g,2_*, using the factor N/(N-1) [13]. In addition, the mean genetic diversity per AFLP locus (*v_mean,2_*) was determined for each species, as an arithmetic average of *v_g,2_* values for all loci.

### Genetic differentiation at AFLP loci

Gene flow, random drift, selection, and mutation create patterns of genetic differentiation, although distinction between these factors by AFLP analysis may be difficult and additional information may be required, such as the use of different genetic markers (e.g. microsatellites). However, extremely high or low genetic differentiation at very few AFLP loci suggests that diversifying forces are acting non-randomly (differential selection or non-recurrent mutation) or uniformly (similar selection regimes in all populations) [38]. Such AFLP loci under natural selection (outlier AFLP loci) were detected using BayeScan v2.1 software [39] [40], which was based on the multinomial Dirichlet model and uses a Reversible Jump Markov Chain Monte Carlo (RJMCMC) algorithm to produce posterior distributions. When the results showed a positive value of the locus-specific component (*α*) and a posterior probability >0.95, we expected differential selection, whereas negative *α* values with posterior probabilities >0.95 indicated possible balancing or purifying selection [39] [41]. The factors of the chain and of the model were as follows: output number of iterations (5,000), thinning interval size (10), pilot runs (20), length of pilot runs (5,000), additional burn in (50,000), prior odds for the neutral model (10), lower boundary for uniform prior on the inbreeding coefficients *F_is_* (0) and the higher boundary for uniform prior on *F_is_* (1).

The differentiation parameter *δ* and its *P(Z≥δ)* value as well as randomly chosen reassignments [38] were applied to test for non-randomly acting diversifying forces at the fourteen locations of the *Picea chihuahuana* tree community sampled.

### Covariation analysis

The relationships between tree species diversity (*ν_sp_*_,*a*_) and the genetic variant ‘2’ (*f_vr_*), genetic diversity (*ν_g,2_*) at each AFLP locus and mean genetic diversity per AFLP locus (*ν_mean,2_*) for each species were measured separately by the covariation (*C*) described by Gregorius et al. [38]. Additionally, *C* values between the number of the four tree species (*DBH* ≥7 cm) per plot (*N*) and the variables *ν_sp_*_,*a*_, *ν_mean,2_*, *ν_g,2_*_,_ and *f_vr_* at putative outlier AFLP loci under differential selection were calculated. Because of the special mathematical structure of the diversity measures used and the frequency of genetic variant ‘2’ (*f_vr_*) at each AFLP locus, it was considered meaningful to look for methods of detecting types of covariation that were monotonous but not necessarily linear. The covariation *C* varied between -1 and 1, where *C* = 1 referred to an entirely positive covariation and *C* = −1 to a strictly negative covariation. When the denominator was zero, *C* was undefined [38]. Formally,
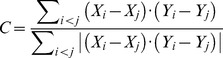
(2)


In order to test the possibility that the observed degrees of covariation *C[ν_sp,a_ xν_g,2_]*, *C[ν_sp,a_ xν_mean,2_]*, *C[ν_sp_ x f_vr_]*, *C[N x ν_sp_]*, *C[N x ν_mean,2_]*, *C[N x ν_g,2_]*, and *C[N x f_vg_]* at putative outlier AFLP loci were only produced by random events rather than directed forces, a one-sided permutation test was performed (here 5,000 permutations) [42].

We preselected only putative outlier AFLP loci under differential selection (false discovery rates [*FDR*] <0.05 and a posterior probability >0.95) with statistical significance of covariation (*C*) between species diversity (*ν_sp_*_,*a*_) and both relative frequency of recessive genetic variant (*f_vr_*) and genetic diversity (*ν_g,2_*) and a positive value of the locus-specific component (*α*). Considering *FDR* as the expected proportion of false positives between outlier markers (Bonferroni correction [43]), both for these outlier AFLP loci and for *C[ν_sp,a_ xν_g,2_]* and *C[ν_sp,_ x f_vr_]*, we selected the outlier AFLP loci with the five highest posterior probabilities. After Bonferroni correction, the new (modified) critical *p* value (significance level*  = 0.01) was calculated by dividing the critical *p* value (here the significance level  = 0.05) by the number of comparisons (hypotheses) (*m* = 5).

If the observed *δ* was larger than 99.6% of imitated *δs* (*i.e. P<*0.004, after Bonferroni correction and six hypotheses (*m* = 6), see below) in the two-sided permutation test, we expected non-randomly acting diversifying forces (differential selection) as causes of the differentiation among the fourteen sampled plots (see details in [38], [44].

## Results

The AFLP primer combination yielded 243 polymorphic bands of 75–450 base pairs across all individuals of *Picea chihuahuana,* while the respective numbers of bands for *Pinus strobiformis*, *Pseudotsuga menziesii*, and *Populus tremuloides* were 250, 207 and 237. Overall, 34–169 AFLP bands were found per *Picea* individual (on average 112), 25–149 per *Pinus* individual (on average 83), 37–140 per *Pseudotsuga* individual (on average 87), and 20–137 per *Populus* individual (on average 64).

Table 2 shows mean genetic diversity per AFLP locus (*ν_mean,2_*) in populations and across all population of the four tree species studied based on the AFLP loci recovered. The highest genetic diversities in *Picea chihuahuana* and *Populus tremuloides* were found in the northern locations, and the highest genetic diversities in *Pinus strobiformis* and *Pseudotsuga menziesii* was found in the southern populations.

**Table 2:** Mean genetic diversity per AFLP locus (*ν_mean,2_*) in *Picea chihuahuana*, *Pinus strobiformis*, *Populus tremuloides,* and in *Pseudotsuga menziesii* populations and across all populations (maximum values in bold).

					*ν_mean,2_*			
Code	Location	*ν_sp,0_*	*ν_sp,2_*	*ν_sp,∞_*	*Picea*	*Pinus*	*Populus*	*Pseudotsuga*
TN	La Tinaja	6	2.75	2.30	1.551	1.237	1.417	
RC	El Ranchito	6	3.85	2.42	1.567	1.596	**1.719**	
CV	El Cuervo	5	3.48	2.56	**1.597**	1.596		
TY	Talayote	7	3.15	2.07	1.564	1.575		1.383
TR	Las Trojas	**8**	4.24	2.70	1.577	1.349	1.510	
VN	El Venado	**8**	4.12	2.41	1.565	1.667		
LQ	La Quebrada	6	3.70	2.67	1.485	1.579	1.414	
PPR	Paraje Piedra Rayada	4	2.29	1.65	1.430	1.417	1.299	1.590
QD	Quebrada de los Durán	5	2.04	1.49	1.459	1.721	1.231	1.540
CB	Cebollitas	7	4.34	2.59	1.525	1.536		1.696
SJ	San José de las Causas	7	**4.46**	**3.00**	1.448	1.654		
SB	Santa Bárbara	9	2.90	1.86	1.439			**1.740**
ACH	Arroyo del Chino	4	3.44	2.73	1.491	1.574		1.685
LP	La Pista	5	1.92	1.49	1.511		1.333	
	across all populations	6.21	3.33	2.28	1.515	1.542	1.418	1.606

**Figure 2:**
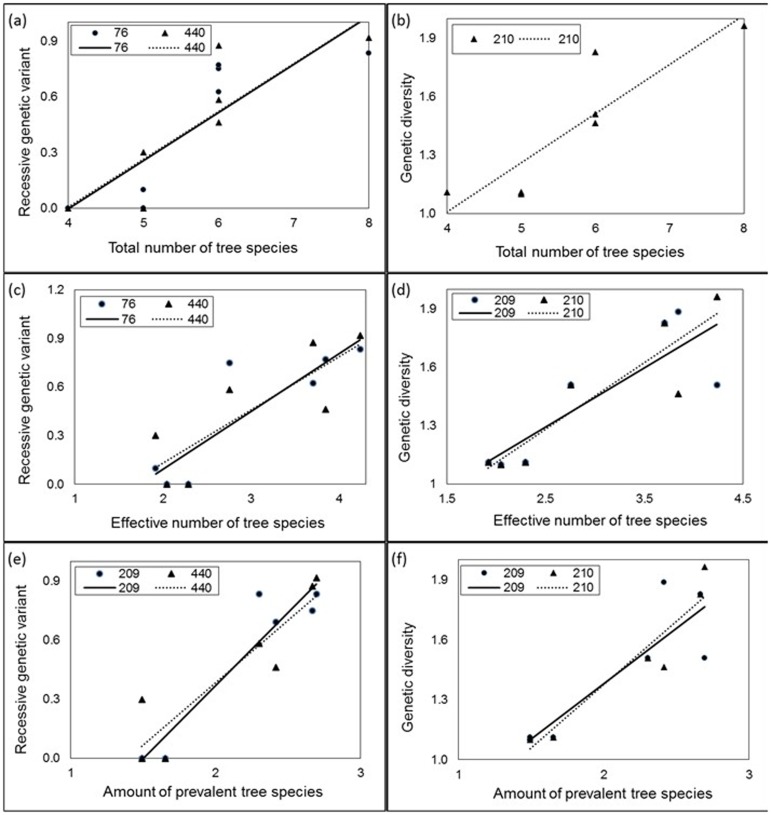
Graphs showing how species diversity (*ν_sp,a_*) is related to recessive genetic variant (*fvr*) and genetic diversity (*ν_g,2_*) at putative adaptive AFLPs in *Populus tremuloides* in the *Picea chihuahuana* tree community.

According to the outlier analysis provided by the BayeScan software, differential selection significantly affected 6.0% of AFLPs in *Picea chihuahuana*, 1.1% in *Pinus strobiformis*, 4.9% of AFLPs in *Populus tremuloides,* and 0.5% in *Pseudotsuga menziesii* (*Table 3*). Before Bonferroni correction of the critical *p* value for covariations (*C[ν_sp,a_ xν_g,2_]* and *C[ν_sp,_ x f_vr_]*), we found only 16 probably adaptive AFLPs in *Populus tremuloides* (76% of all adaptive AFLPs in *Populus tremuloides*) associated with tree species diversity. *C[ν_sp,a_ xν_g,2_]* and *C[ν_sp,_ x f_vr_]* were positive in *Populus tremuloides* (*Table 4* *,* *Figure 2*).

**Table 3:** Candidate AFLP loci under differential selection (False Discovery Rate (FDR) <0.05 and posterior probability >0.95) in three tree species in the *Picea chihuahuana* tree species community. *α* is the locus-specific component.

Species	AFLP	(α>0)	Posterior probabilities	FDR
*Picea chihuahuana*	122	0.937	0.992	0.002
	226	1.160	1.000	0.000
	227	1.195	1.000	0.000
	267	0.987	0.991	0.003
	331	0.928	0.984	0.007
	337	1.133	1.000	0.000
	341	1.172	1.000	0.000
	345	1.048	0.999	0.000
*Pinus strobiformis*	128	1.109	0.977	0.013
	291	1.258	0.995	0.005
	434	1.207	0.990	0.008
*Populus tremuloides*	76	1.627	0.991	0.004
	88	1.724	0.993	0.003
	89	1.665	0.994	0.003
	150	1.704	0.996	0.001
	156	1.727	0.995	0.003
	160	1.662	0.995	0.002
	165	1.667	0.991	0.004
	169	1.950	1.000	0.000
	179	1.611	0.984	0.005
	184	1.672	0.994	0.003
	188	1.622	0.986	0.005
	190	2.177	1.000	0.000
	193	1.592	0.991	0.004
	198	1.783	0.998	0.001
	208	1.530	0.978	0.006
	209	1.926	1.000	0.000
	210	1.820	0.999	0.000
	220	1.730	0.996	0.002
	249	1.692	0.996	0.002
	253	1.766	0.996	0.002
	440	1.522	0.956	0.008
*Pseudotsuga menziesii*	110	1.529	0.986	0.014

**Table 4:** Covariation (*C[ν_sp,a_ xν_g,2_]* and *C[ν_sp,a_ x f_vr_]*) between species diversity (*ν_sp,a_*) and relative frequency of genetic variant ‘2’ (*f_vr_*) as well as genetic diversity (*ν_g,2_*) at the AFLP loci under differential selection.

*Diversity*	*Species*	*f_vr_*			*v_g,2_*		
		*AFLP*	*C*	*P(Z≥C)*	*AFLP*	*C*	*P(Z≥C)*
*ν_sp,0_*	*Populus tremuloides*	76	0.99	0.005	88	0.99	0.041
		156	0.99	0.023	150	0.90	0.039
		169	0.95	0.040	179	0.96	0.035
		184	0.98	0.009	188	0.99	0.042
		198	0.99	0.034	210*	0.99	0.009
		209	0.99	0.014	220	0.96	0.019
		220	0.99	0.012			
		249	0.99	0.009			
		253	0.99	0.019			
		440	0.99	0.005			
*ν_sp,2_*	*Populus tremuloides*	76	0.98	0.009	76	0.83	0.041
		150	0.93	0.039	160	0.92	0.035
		156	0.95	0.020	165	0.97	0.029
		165	0.99	0.022	184	0.96	0.018
		179	0.92	0.039	188	0.99	0.017
		184	0.87	0.038	190	0.94	0.018
		198	0.89	0.039	209	0.95	0.022
		209	0.97	0.018	210	0.98	0.012
		210	0.86	0.043	220	0.95	0.013
		220	0.89	0.027	249	0.91	0.039
		249	0.94	0.027			
		253	0.89	0.033			
		440	0.95	0.013			
*ν_sp,∞_*	*Populus tremuloides*	76	0.98	0.015	76	0.94	0.013
		156	0.90	0.044	160	0.98	0.011
		184	0.93	0.019	184	0.98	0.010
		209*	**0.99**	**0.008**	209	0.96	0.013
		249	0.98	0.013	210*	**0.99**	**0.005**
		440	0.98	0.005	220	0.93	0.020
					249	0.92	0.037

All C values were statistically significant before Bonferroni correction.

Note: * statistically significant after Bonferroni correction (in **bold**).

After Bonferroni correction of the critical *P* value for *C[ν_sp,a_ xν_g,2_]* and *C[ν_sp,_ x f_vr_]*, positive covariations (*C*) between species diversity (*ν_sp_*_,*a*_) and genetic variant ‘2’ (*f_vr_*) as well as genetic diversity (*ν_g,2_*) were observed at the putatively adaptive AFLPs 209 and 210 in *Populus tremuloides* (*Tables 2* and *3*).

We found moderately positive covariation (*C*) between species diversities (*ν_sp_*_,*a*_) and mean genetic diversity per AFLP locus (*ν_mean,2_*) for *Picea chihuahuana* and moderately to strongly positive covariation for *Pseudotsuga menziesii* and *Populus tremuloides*; however, these values were not significant after Bonferroni correction (*Table 5*).

**Table 5:** Covariation (*C*) between species diversity (*ν_sp_*_,*a*_) mean genetic diversity per AFLP locus (*ν_mean,2_*) and its P(Z≥C) value in *Picea chihuahuana*, *Pinus strobiformis*, *Populus tremuloides,* and in *Pseudotsuga menziesii*.

Tree species	*a*	*C[ν_sp,a_ x ν_mean,2_]*	*P(Z≥C)*
*Picea chihuahuana*	0	+0.23	0.288
	2	+0.51	0.089
	∞	+0.45	0.120
*Pinus strobiformis*	0	−0.03	0.486
	2	+0.19	0.350
	∞	−0.02	0.498
*Populus tremuloides*	0	+0.83	0.058
	2	+0.96	0.011
	∞	+0.90	0.030
*Pseudotsuga menziesii*	0	+0.24	0.410
	2	+0.51	0.237
	∞	+0.51	0.249

Finally, the covariation (*C*) between number of the tree species per plot (*N*) and *ν_sp_*_,*2*_ and *ν_g_*_,*2*_ at the putative adaptive AFLP 210 was significantly positive for *Populus tremuloides* before Bonferroni correction (*C[N x ν_sp,2_]*  = +0.95, *P* = 0.02, *C[N x ν_g,2, 210_]*  = +0.90, *P* = 0.03). However, the relationships between *N* and *ν_mean,2_*, *ν_g,2_* at AFLP locus 209 and *f_vg_* at the AFLP loci 209 and 210 were moderate (*C* = +0.65, +0.73, −0.57 and −0.61) and statistically non-significant (*P* = 0.18, 0.14, 0.19 and 0.17) for *Populus tremuloides*.

The differentiation (*δ*) value for the fourteen sampled plots of *Picea chihuahuana* tree community was 0.583, with a *P(Z≥δ)* value lower than 0.000001.

## Discussion and Conclusions

Assuming that the mapped markers used in this study reflect the whole genome, 0.5–6.0% of AFLP loci affected by possible differential selection were detected in the four tree species sampled from the *Picea chihuahuana* tree community in the Sierra Madre Occidental, Mexico. Previous studies of interspecific variation in *Quercus petraea* and *Quercus robur* [45], *Picea abies* [46], *Fagus sylvatica* [47], and *Pinus monticola* [48] in other parts of the world showed similar proportions of putative genetic AFLP loci caused by selection (12.7%, 2.5–3.3%, 0.4%, and 12.0%, respectively). On a worldwide scale, the discovery of probably adaptive loci has increasingly been reported across ecological gradients in various species [48].

The results of this study clearly show that species diversity (*ν_sp,a_*) was positively and significantly only related to genetic variant ‘2’ (*f_vr_*) and genetic diversity (*ν_g,2_*) at two putatively adaptive AFLPs in *Populus tremuloides* in the *Picea chihuahuana* tree community under study (*Table 4*). A large proportion of putative adaptive AFLP (76%) in *Populus tremuloides* was always positively, but statistically non-significantly, associated with species diversity (*Table 4*). Together these results demonstrated that *Populus tremuloides* evolve within a community context.

The number of *Populus tremuloides* trees per plot (*N*) explained the positive relationship between tree species diversity and mean (multilocus) genetic diversity per AFLP locus because population size has been positively related to mean genetic diversity [66]. The *Populus tremuloides* trees were more frequent in locations of higher tree species diversity. The highest tree species diversity and tree density were found in the most humid and coldest climate on the Sierra Madre Occidental in Durango [67], the optimal climate conditions for *Populus tremuloides* in forests of Durango [68]. However, we cannot explain why *N* was positively correlated with genetic diversity by differential selection (at the putatively adaptive AFLP 210). Thus, we can only speculate as to why the genetic structure at the putative adaptive AFLP 209 and 210 of *Populus tremuloides* was strongly correlated with species diversity in the tree community under study. This may have been due to the dioecious nature, mating system [49], [50], low genome size (∼550 Mbp) [51]), and/or the pioneering strategy of the quaking poplar, which is the most widely distributed tree species in North America [52] and displays higher genetic adaptation to different environmental conditions than species of other genera [53] [54].

The relationship between the two levels of diversity was positive for detected putatively adaptive AFLP (*Table 4*). Thus, the genetic [29] [47] [55] [56] [57] and species structures [58] in the tree community were possibly simultaneously adapted to a combination of ecological or environmental factors [6] [3] [31]. This conclusion appears plausible because the values for differentiation (*δ*) among the fourteen sampled locations under study in the *Picea chihuahuana* tree community also indicate a strong effect of non-randomly acting diversifying forces (differential selection) on species diversity [44]. However, genetic differentiation may also be directly affected by the differential genetic response [59] to competition with other tree species [60].

The positive but non-significant relationships between tree species diversity and the mean genetic diversity per AFLP locus (including all AFLPs) found in three tree species under study (*Table 5*) implied selection (*Table 3*) that may affect higher multilocus genetic diversity and thus drive individual specialization [61]. The present observations also support the findings of [7], i.e. that the decrease in species diversity is lower in communities with higher within-population genetic diversity. Perhaps the locations that are rich in both tree species and genetic diversity (*Table 2*) [27] [69] were the oldest, i.e. they spent more time to enrich new genetic variants and species [62].

The present findings indicate the existence of correlations between genetic and species diversity as the two most important levels of biodiversity [2] and that interactions between genetic variants and species diversity may be crucial in shaping tree communities [30] [59] [63] [64].

Thus, the present results may contribute to a better understanding of the concurrence of evolutionary and ecological processes for determining community structure and dynamics [33] [34] and thus help to develop preservation and conservation strategies for this rare tree species community [33]. However, further study is needed to detect the complex variable local characteristics that influence both genetic and species diversity in the *Picea chihuahuana* tree community [31].

## Supporting Information

Data S1 **Raw data used in this work.**
(XLSX)

## References

[1] Piñero D, Caballero-Mellado J, Cabrera-Toledo D, Canteros CE (2008) La diversidad genética como instrumento para la conservación y el aprovechamiento de la biodiversidad: estudios en especies mexicanas, en Capital natural de México, vol. I: Conocimiento actual de la biodiversidad. CONABIO, México p 437–494.

[2] Bergmann F, Gregorius HR, Kownatzki D, Wehenkel C (2013) Different diversity measures assess species-genetic diversity relationships differently: A marker-based case study in forest tree communities. Silvae Genet 62 1–2: 25–38.

[3] Vellend M, Geber MA (2005) Connections between species diversity and genetic diversity. Ecol. Lett 8: 767–781.

[4] Wehenkel C, Bergmann F, Gregorius HR (2006) Is there a trade-off between species diversity and genetic diversity in forest tree communities? Plant Ecol 185: 151–161.

[5] Gibson DJ, Allstadt AJ, Baer SG, Geisler M (2012) Effects of foundation species genotypic diversity on subordinate species richness in an assembling community. Oikos 121: 496–507.

[6] Vellend M (2005) Species diversity and genetic diversity: parallel processes and correlated patterns. The American Naturalist 166, 199–215. Ecol. Lett 8: 767–781. 10.1086/431318 16032574

[7] Booth RE, Grime JP (2003) Effects of genetic impoverishment on plant community diversity. J. Ecol 91: 721–730.

[8] Karlin AA, Guttman SI, Rathbun SL (1984) Spatial autocorrelation analysis of heterozygosity and geographic distribution in population of *Desmognathus fuscus* (Amphibia: Plethodontidae). Copeia 1984: 341–354.

[9] Whittaker RH (1972) Evolution and Measurement of Species Diversity. Taxon 21: 213–251.

[10] Shannon CE (1948) A mathematical theory of communication. The Bell System Technical Journal 27: 379–656 and –

[11] Simpson EH (1949) Measurement of diversity. Nature 163: 688.

[12] Hill MO (1973) Diversity and evenness: a unifying notation and its consequences. Ecology 54: 427–432.

[13] Gregorius HR (1978) The concept of genetic diversity and its formal relationship to heterozygosity and genetic distance. Math. Biosci 41: 253–432.

[14] Zyczkowski K (2003) Rényi Extrapolation of Shannon Entropy. Open Sys & Information Dyn 10: 297–310.

[15] Jost L (2006) Entropy and diversity. Oikos 113: 363–375.

[16] Jost L (2007) Partitioning diversity into independent alpha and beta components. Ecology 88: 2427–2439. 18027744 10.1890/06-1736.1

[17] Gregorius HR (2010) Linking diversity and differentiation. Diversity 2: 370–394 doi:10.3390/d2030370

[18] Norma Oficial Mexicana (2010) NOM-059-ECOL-2010. Protección ambiental -Especies nativas de México de flora y fauna silvestres- Categorías de riesgo y especificaciones para su inclusión, exclusión o cambio. Lista de especies en riesgo. Diario Oficial de la Federación (Segunda sección): 1–77.

[19] IUCN Red List of Threatened Species (2013) Available: http://www.iucnredlist.org/details/32479/0 Accessed 2013 Apr 8.

[20] Ledig FT, Mapula LM, Bermejo VB, Reyes HV, Flores-López C, et al (2000) Locations of endangered spruce populations in Mexico and the demography of *Picea chihuahuana* . Madroño 47: 71–88.

[21] Sáenz-Romero C, Rehfeldt GE, Crookston NL, Duval P, St-Amant R, et al (2010) Spline models of contemporary, 2030, 2060 and 2090 climates for Mexico and their use in understanding climate-change impacts on the vegetation. Clim. Chang 102: 595–623.

[22] Narváez FR (1984) Contribución al Conocimiento de la Ecología de Picea chihuahuana. Tesis profesional (Biología), Universidad Autónoma de Nuevo León, Facultad de Ciencias Biológicas. México.

[23] Sánchez CJ (1984) *Picea chihuahuana*, conífera en Peligro de Extinción. Cienc. Forest 9: 51–63.

[24] Gordon AG (1968) Ecology of *Picea chihuahuana* Martínez. Ecology 49: 880–896.

[25] Ledig FT, Jacob-Cervantes V, Hodgskiss PD, Eguiluz-Piedra T (1997) Recent evolution and divergence among populations of a rare Mexican endemic, Chihuahua spruce, following Holocene climatic warming. Evolution 51: 1815–1827. 28565106 10.1111/j.1558-5646.1997.tb05105.x

[26] Quiñones-Pérez CZ, Silva-Flores R, Wehenkel C (2012) Ecology of the Mexican *Abies durangensis* Martínez. Kastamonu University Journal of Forestry Faculty 12(3): 180–184.

[27] Jaramillo-Correa JP, Beaulieu J, Ledig FT, Bousquet J (2006) Decoupled mitochondrial and chloroplast DNA Population structure reveals holocene collapse and population isolation in a threatened mexican-endemic conifer. Mol. Ecol 15: 2787–2800. 16911200 10.1111/j.1365-294X.2006.02974.x

[28] Wehenkel C, Martínez-Guerrero JH, Pinedo-Álvarez A, Carrillo A (2012) Adaptive genetic differentiation in *Picea chihuahuana* M. caused by different copper concentrations in the top soil. Forstarchiv 83: 48–51.

[29] Wehenkel C, Sáenz-Romero C (2012) Estimating genetic erosion using the example of *Picea chihuahuana* Martínez. Tree Genet. Genomes 8: 1085–1094.

[30] Quiñones-Pérez CZ, Sáenz-Romero C, Wehenkel C (2014) Influence of neighbouring tree species on AFLP variants of endangered *Picea chihuahuana* populations on the Sierra Madre Occidental, Northeastern México. Pol. J. Ecol 62(1): 69–79.

[31] Wehenkel C, Sáenz-Romero C, Jaramillo-Correa JP (2014) Estimating genetic erosion in threatened conifers: the example of *Picea chihuahuana* Martínez, Chapter 15: 20 pages in Ahuja, M. R. and Jain, S.M. (eds.): Genetic Erosion and Biodiversity, Springer SBM, The Netherlands, in press.

[32] Ledig TL, Rehfeldt GE, Sáenz-Romero C, Flores-López C (2010) Projections of suitable habitat for rare species under global warming scenarios. Am. J. Bot 97 (6): 970–987. 10.3732/ajb.0900329 21622467

[33] Antonovics J (1992) Toward community genetics. In: Fritz RS, Simms EL (eds) Plant resistance to herbivores and pathogens: ecology evolution and genetics. University of Chicago Press, Chicago, pp 429–449.

[34] Antonovics J (2003) Toward community genomics? Ecology 84: 598–601.

[35] Vos PR, Hogers, Bleeker M (1995) AFLP: a new concept for DNA fingerprinting. Nucleic Acids Res 23: 4407–4414. 7501463 10.1093/nar/23.21.4407 PMC307397

[36] Krauss SL (2000) Accurate gene diversity estimates from amplified fragment length polymorphism (AFLP) markers. Mol. Ecol 9: 1241–1245. 10972764 10.1046/j.1365-294x.2000.01001.x

[37] Bonin A, Bellemain E, Bronken EP, Pompanon F (2004) How to track and assess genotyping errors in population genetics studies. Mol. Ecol 13: 3261–3273. 15487987 10.1111/j.1365-294X.2004.02346.x

[38] Gregorius HR, Degen B, König A (2007) Problems in the analysis of genetic differentiation among populations a case study in *Quercus robur* . Silvae Genet 56: 190–199.

[39] Foll M, Gaggiotti OE (2008) A genome scan method to identify selected loci appropriate for both dominant and codominant markers. A Bayesian perspective Genetics 180: 977–993. 10.1534/genetics.108.092221 PMC2567396 18780740

[40] Fischer MC, Foll M, Excoffier L, Heckel G (2011) Enhanced AFLP genome scans detect local adaptation in high-altitude populations of a small rodent (*Microtus arvalis*). Mol. Ecol 20: 1450–1462. 21352386 10.1111/j.1365-294X.2011.05015.x

[41] Foll M, Fischer MC, Heckel G, Excoffier L (2010) Estimating population structure from AFLP amplification intensity. Mol. Ecol 19: 4638–4647. 20874760 10.1111/j.1365-294X.2010.04820.x

[42] Manly BFJ (1997) Randomization bootstrap and Monte Carlo methods in biology - Chapman and Hall. London, p 399.

[43] Hochberg Y (1988) A sharper Bonferroni procedure for multiple tests of significance. Biometrika 75 (4): 800–802.

[44] Wehenkel C, Corral-Rivas JJ, Castellanos-Bocaz HA (2010) Is there selection by species diversity in *Picea abies* L.? Plant Ecol 208(1): 47–54.

[45] Scotti-Saintagne C, Mariette S, Porth I (2004) Genome scanning for interspecific differentiation between two closely related oak species [*Quercus robur* L. and *Q. petraea* (Matt.) Liebl.]. Genetics 168: 1615–1626. 15579711 10.1534/genetics.104.026849 PMC1448783

[46] Acheré VJ, Favre M, Besnard G, Jeandroz S (2005) Genome organization of molecular differentiation in Norway spruce (*Picea abies*). Mol. Ecol 14: 3191–3201. 16101784 10.1111/j.1365-294X.2005.02646.x

[47] Jump AS, Hunt JM, Peñuelas J (2006) Rapid climate change related growth decline at the southern range edge of *Fagus sylvatica* . Mol. Ecol 12(11): 2163–2174.

[48] Richardson BA, Rehfeldt GE, Kim MS (2009) Congruent climate-related genecological responses from molecular markers and quantitative traits for western white pine (*Pinus monticola*). Int. J. Plant. Sci 170: 1120–1131.

[49] Chan FJ, Wong RM (1989) Reestablishment of native riparian species at an altered high elevation site. In: Abell, Dana L, technical coordinator. Proceedings of the California riparian systems conference: Protection, management, and restoration for the 1990's; 1988 September 22–24; Davis, CA. Gen. Tech. Rep. PSW-110. Berkeley, CA: U.S. Department of Agriculture, Forest Service, Pacific Southwest Forest and Range Experiment Station: 428–435. [13771].

[50] Wang XZ, Curtis PS (2001) Gender-specific response of *Populus tremuloides* to atmospheric CO_2_ enrichment. New Phytol 150: 675–684.

[51] Wullschleger SD, Jansson S, Taylor G (2002) Genomics and forest biology: *Populus* emerges as the perennial favorite. Plant Cell 14: 2651–2655. 12417692 10.1105/tpc.141120 PMC540295

[52] Worrall JJ, Rehfeldt GE, Hamann A, Hogg EH, Marchetti SB, et al (2013) Recent declines of *Populus tremuloides* in North America linked to climate. For. Ecol. Manage 299: 35–51.

[53] Jelinski DE (1992) Genetic diversity and spatial subdivision of *Populus tremuloides* (Salicaceae) in a hetereogeneus landscape. Am. J. Bot 79 (7): 728–736.

[54] Lindroth RL, St. Clair SB (2013) Adaptations of quaking aspen (*Populus tremuloides* Michx.) for defense against herbivores. For. Ecol. Manage 299: 14–21.

[55] Zangerl AR, Bazzaz FA (1984) The partitioning between two phosphoglucoisomaerase genotypes in amaran thus retroflexus. Ecology 65: 218–222.

[56] Lumaret R (1984) The role of polyploidy in the adaptive significance of polymorphism at the GOT 1 locus in the *Dactylis glomerata* complex. Heredity 52: 153–169.

[57] Kelly CK, Chase M, Fay MF, de Bruijn A, Woodward FI (2003) Temperature-based population segregation in birch. Ecol. Lett 6: 87–89.

[58] Dupre C, Wessberg C, Diekmann M (2002) Species richness indeciduous forests: effects of species pools and environmental variables. J. Veg. Sci 13: 505–516.

[59] Wehenkel C, Bergmann F, Gregorius HR (2007) Interactions between genetic structures and species composition in forest tree communities. Silvae Genet 56(3–4): 101–110.

[60] Vavrek MC (1998) Within-population genetic diversity of *Taraxacum officinale* (Asteraceae). Differential genotype response and effect on interspecific competition. Am. J. Bot 85: 947–954. 21684978

[61] Aarssen LW, Turkington R (1985) Biotic specialization between neighbouring genotypes in *Lolium perenne* and *Trifolium repens* from a permanent pasture. J. Ecol 73: 605–614.

[62] Vellend M (2004) Parallel effects of land-use history on species diversity and genetic diversity of forest herbs. Ecology 85: 3043–3055.

[63] Callaway RM, Walker LR (1997) Competition and facilitation: a synthetic approach to interactions in plant communities. Ecology 78: 1958–1965.

[64] Brooker RW (2006) Plant–plant interactions and environmental change. New Phytol 171: 271–289. 16866935 10.1111/j.1469-8137.2006.01752.x

[65] Mendoza-Maya E, Espino-Espino J, Quiñones-Pérez CZ, Flores-López C, Wehenkel C, et al.. (2014) Propuesta de conservación de tres especies mexicanas de picea en peligro de extinción. In press.

[66] Frankham R (1996) Relationship of genetic variation to population size in wildlife. Conserv. Biol 10(6): 1500–1508.

[67] Silva-Flores R, Perez-Verdin G, Wehenkel C (2014) Relationship between diversity of tree species and climatic factors in the Sierra Madre Occidental, Mexico. PLOS One. In Press. 10.1371/journal.pone.0105034 PMC4134238 25127455

[68] Martínez-Antúnez P, Wehenkel C, Hernández-Díaz JC, Gonzáles-Elizondo M, Corral-Rivas JJ, et al (2013) Effect of climate and physiography on the density of tree and shrub species in Northwest Mexico. Pol. J. Ecol 61(2): 283–295.

[69] Quiñones-Pérez CZ, Simental-Rodríguez SL, Saenz-Romero C, Jaramillo-Correa JP, Wehenkel C (2014) Spatial genetic structure in the very rare and species-rich *Picea chihuahuana* tree community (Mexico). Silvae Genet., In Press.

[70] Kuchma O, Vornam B, Finkeldey R (2011) Mutation rates in Scots pine (*Pinus sylvestris* L.) from the Chernobyl exclusion zone evaluated with AFLP and microsatellite markers. Mutat. Res 725: 29–35. 21782970 10.1016/j.mrgentox.2011.07.003

